# Indications of early intubation for patients with inhalation injury

**DOI:** 10.1002/ams2.269

**Published:** 2017-03-06

**Authors:** Shinya Onishi, Akinori Osuka, Yuichi Kuroki, Masashi Ueyama

**Affiliations:** ^1^ Department of Trauma, Critical Care Medicine and Burn Center Japan Community Health Care Organization Chukyo Hospital Nagoya AICHI Japan

**Keywords:** Bronchoscopy, diagnosis, smoke inhalation injury

## Abstract

**Aim:**

For patients with inhalation injury, the indications for early intubation are diverse. The purpose of this study was to identify the most reliable symptoms, physical findings, and medical examinations with which to determine the indications for early intubation in patients with inhalation injury.

**Methods:**

We retrospectively collected patient data from medical records. Collected data included age, sex, burn size, symptoms, physical findings, carboxyhemoglobin levels (COHb), and bronchial wall thickness (BWT) determined from chest computed tomography images. We analyzed the relationships between these findings and the early intubation. We performed fiberoptic bronchoscopy in all patients, and analyzed the relationships between bronchoscopic severity and other findings.

**Results:**

Of the 205 patients, 80 patients were diagnosed as having inhalation injury, and 34 patients were intubated. Burn size, facial burns, neck burns, use of accessory respiratory muscles, and COHb seemed to be related with intubation, whereas singed nasal hair was not. If the patients suffered ≥27% total body surface area burn and BWT ≥3.5 mm, the positive predictive value for early intubation was 1.00. If the patients suffered smaller cutaneous burn without neck burn, and their COHb <4.0%, the negative predictive value for early intubation was 0.97. Fiberoptic bronchoscopy findings from above the glottis were mainly related with patients’ symptoms. Findings from below the glottis were mainly related with BWT and COHb.

**Conclusions:**

Patients’ symptoms, especially use of accessory respiratory muscles, are reliable, and BWT and COHb are also useful tools, for determining the indication for early intubation.

## Introduction

Inhalation injury is a life‐threatening injury. The mortality of inhalation injury without cutaneous burn injury is approximately 10%, whereas with cutaneous burn injury, the mortality increases more than twice.^1^, ^2^ Inhalation injury increases fluid requirements^3^ and the incidence of pneumonia.^4^ In patients with inhalation injury, early intubation is indicated if upper airway patency is in danger. Intubation is also required when patients are in shock, with impaired oxygenation, or coma. In such urgent situations, the necessity of intubation is obvious. However, for patients with suspected inhalation injury, such as patients with singed nasal hair or the presence of carbonaceous sputum, the necessity of intubation is still undefined. The clinical diagnosis of inhalation injury can be inexact because it relies on the constellation of a history of fire in an enclosed space, elevated carboxyhemoglobin on admission, and the presence of soot sputum.^5^, ^6^ The severity of inhalation injury can be difficult to classify on their admission to the burn center. Researchers have tried to characterize the severity of inhalation injury, using computed tomography (CT) to identify anatomical changes,^7^, ^8^ and the calculation of V/Q ratios to assess gas exchange.^9^ Others have investigated the possibility of biomarkers in plasma or alveolar fluid to determine the severity of inhalation injury.^10^, ^11^, ^12^ Bronchoscopy is a useful tool to diagnose inhalation injury that allows us to see the patient's bronchial surface directly, and to collect alveolar lavage.^13^, ^14^, ^15^, ^16^, ^17^, ^18^ Masanes *et al*.^17^ found that inhalation injury could be diagnosed by fiberoptic bronchoscopy (FOB) in burn patients who otherwise had no symptoms. However, Liffner *et al*.^18^ reported that their scoring system for grading the severity of inhalation injury by bronchoscopy did not correlate with the incidence of acute respiratory distress syndrome. Similarly, although it is generally recognized that inhalation injury increases fluid resuscitation needs in burn patients, Endorf and Gamelli found no correlation of the severity of bronchoscopic findings with fluid resuscitation requirements.^13^

Working in the burn center, we sometimes experience unnecessary intubated patients transferred from other hospitals. The intubation requires more sedation, which requires more resuscitation fluids, and could cause burn‐induced compartment syndromes^19^ or ventilator‐associated respiratory complications. In this paper, we wish to distinguish the most reliable findings with which to determine the indications for early intubation in patients with inhalation injury.

## Methods

We retrospectively collected patient data from patients admitted to our burn center from January 1, 2012 to March 31, 2014. Inclusion criteria were inhalation injury diagnosed by FOB. Patients transferred more than 24 h after injury or patients whose age under 18 years were excluded. From the medical records, we collected patient data such as age, sex, percentage of total body surface area (TBSA) of second‐ and third‐degree burns, symptoms and physical findings (singed nasal hair, hoarseness, facial or neck burns, use of accessory respiratory muscles, and noisy breath sounds), and carboxyhemoglobin (COHb) levels. Bronchial wall thickness (BWT) was measured from chest CT images. First, we analyzed the relationships between these findings and early intubation. Next, we analyzed the relationships between FOB severity and other findings. We developed the original FOB grading system shown in Table 1, with which we evaluated the severity of inhalation injury in both the upper and lower airway. We diagnosed inhalation injury based on M (mild) or S (severe) grading in our scale, and if the patients were evaluated as S for any bronchoscopic finding, we considered them at high risk for inhalation injury and intubated them. We also intubated due to other reasons that the emergency room (ER) physicians considered significant, such as coma or extensive burn injury. We have reported that this classification can be a useful tool for the grading of inhalation injury above the glottis.^20^ For inhalation injury of the lower glottis, Abbreviated Injury Scale (AIS) grades of three or more are considered S grades in our scale. We defined early intubation as intubation required within 12 h after injury. If we could extubate the patients within 24 h after injury, we defined that they were unnecessary intubated.

**Table 1:** Our bronchoscopic grading of inhalation injury

Our grade	Findings	AIS grade	Findings
L−	Absence of carbonaceous deposits, erythema, edema, bronchorrhea, or obstruction.		
Lm	Minor or patchy areas of erythema, carbonaceous deposits in laryngeal area.		
Ls	1) Epiglottic blister or bullae.		
	2) Swelling of arytenoid region extends to the aryepiglottic fold.		
	3) The length of the center of the glottic space when the vocal cords open maximally is shorter than the width of the center of the arytenoid region.		
	4) The vocal cords are obscured because the vestibular folds are swollen.		
	5) The glottis does not open sufficiently due to marked vocal cord swelling (any or combination).		
B−	Absence of carbonaceous deposits, erythema, edema, bronchorrhea, or obstruction.	Grade 0 (no injury)	Absence of carbonaceous deposits, erythema, edema, bronchorrhea, or obstruction
Bm	Hyperemia and/or edema of the bronchus with evidence of blood flow (presence of visible vessels through the mucosa).	Grade 1 (mild injury)	Minor or patchy areas of erythema, carbonaceous deposits in proximal or distal bronchi (any or combination).

		Grade 2 (moderate injury)	Moderate degree of erythema, carbonaceous deposits, bronchorrhea, with or without compromise of the bronchi (any or combination).
Bs	Severe edema, ulcer, necrosis of the bronchus without evidence of blood flow (pale mucosa without visible vessels), bronchorrhea, bronchial obstruction (any or combination).	Grade 3 (severe injury)	Severe inflammation with friability, copious carbonaceous deposits, bronchorrhea, bronchial obstruction (any or combination).
		Grade 4 (massive injury)	Evidence of mucosal sloughing, necrosis, endoluminal obliteration (any or combination).

Data are presented as number of cases or median (interquartile range). Fisher's exact test or Wilcoxon's rank‐sum test was used to compare the patient characteristics and findings shown in Table 2. Positive and negative prediction values for early intubation were determined using the accuracy of qualitative test. Cut‐off values for continuous variables were determined by receiver–operating characteristic curve analysis. Then classification and regression tree analysis was carried out to find out the most impact factors for early intubation. The relationships between FOB severities and categorical data were tested using Fisher's exact test and continuous variables were analyzed by the Kruskal–Wallis test. For each test, differences were considered statistically significant at a *P*‐value of <0.05. Data were analyzed using JMP Pro for Windows version 11.0.0 (SAS Institute, Cary, NC, USA) and R for Windows (http://www.jichi.ac.jp/saitama-sct/SaitamaHP.files/statmed.html).

**Table 2:** Characteristics of patients with inhalation injury and findings at first presentation

	Total	Intubation (+)	Intubation (−)	*P* value
	*n* = 80	*n* = 34	*n* = 46	
Age, years	59 (41, 74)	58 (43, 72)	63 (41, 72)	0.90
Gender, male/female	41/39	18/16	23/23	0.82
TBSA burned, %	9.5 (0.3, 30)	30 (11, 45)	3.5 (0, 13)	<0.001
Facial burns	48	26	22	0.02
Neck burns	32	21	11	0.001
Singed nasal hair	51	24	27	0.35
Hoarseness	24	11	13	0.81
Use of accessory respiratory muscles	5	5	0	0.012
Noisy breath sounds	9	6	3	0.16
BWT, mm	3.3 (2.7, 4.0)	3.5 (2.0, 11.0)	3.0 (1.2, 4.9)	0.06
COHb, %	3.0 (1.2, 8.4)	5.3 (2.5, 20.0)	2.2 (1.0, 4.1)	0.001
Time to intubation after injury, min	−	67.5 (46, 137)	−	−

Data are expressed as number of cases or median (interquartile range) or numbers.

Fisher's exact test or Wilcoxon rank–sum test was used to compare the intubation (+) group with the no intubation (−) group.

BWT, bronchial wall thickness; COHb, carboxyhemoglobin level; TBSA, total body surface area.

This study was approved by the Institutional Review Board of the Japan Community Health care Organization Chukyo Hospital (Registration No. 2016013).

## Results

Of the 205 patients admitted our burn center, 80 patients suffered inhalation injury, and 34 patients were intubated within 12 h following injury. Patient characteristics and physical findings at the initial presentation to the ER are shown in Table 2. The percentage TBSA of second‐ and third‐degree burns, facial burns, neck burns, use of accessory respiratory muscles, and COHb levels were significantly related with early intubation (*P* < 0.05), whereas singed nasal hair and hoarseness were not significantly related (*P* = 0.35 and 0.81, respectively). The indications for intubation are shown in Table 3. For safe transfer by helicopter, five patients were intubated. All these patients suffered facial burn and 80% of them had neck burns. Three patients were ultimately intubated more than 12 h after burn injury. At their initial presentation in the ER, they were diagnosed as having mild inhalation injury. However, they were intubated at 16, 22, and 52 h after initial burn injury because of progressing upper airway edema. All three of these patients had facial and neck burns. We could not extubate any patients within 24 h.

**Table 3:** Indications for early intubation in patients with inhalation injury

	*n* = 34
Ls	9
Bs	5
LsBs	3
Coma	4
Extensive burn	6
Neck burn	1
Facial burn	6
Safe transfer	5

Bs, severe edema, ulcer, necrosis of the bronchus without evidence of blood flow (pale mucosa without visible vessels), bronchorrhea, bronchial obstruction (any or combination). Ls, (i) epiglottic blister or bullae; (ii) swelling of arytenoid region extends to the aryepiglottic fold; (iii) the length of the center of the glottic space when the vocal cords open maximally is shorter than the width of the center of the arytenoid region; (iv) the vocal cords are obscured because the vestibular folds are swollen; (v) the glottis does not open sufficiently due to marked vocal cord swelling (any or combination).

**Figure 1:**
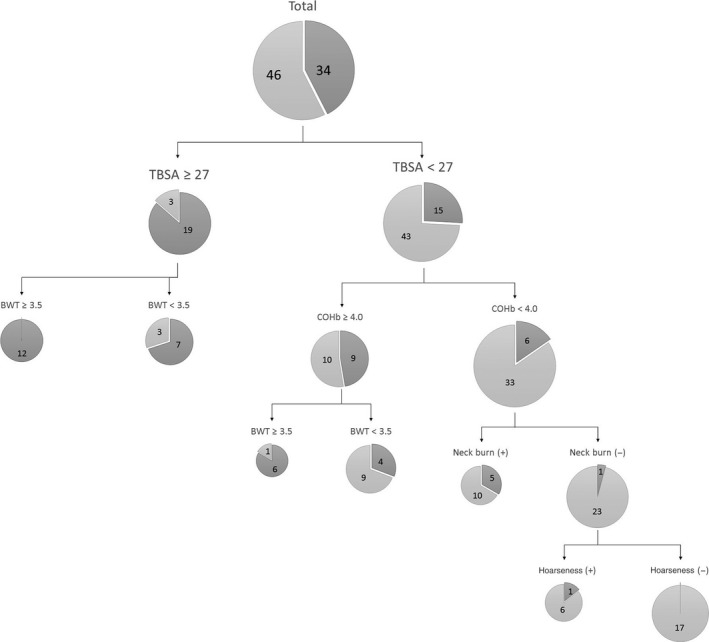
Charts showing early intubation for patients with inhalation injury partitioned by the symptoms and physical findings using a classification and regression tree analysis. BWT, bronchial wall thickness; COHb, carboxyhemoglobin level; TBSA, total body surface area.

The positive and negative predictive values of each parameter for early intubation are shown in Table 4. The cut‐off values 27% for TBSA, 3.5 mm for BWT, and 4.0% for COHb were computed. The highest positive predictive value was use of accessory respiratory muscles, and the highest negative predictive value was TBSA ≥ 27%. Classification and regression tree analysis determined that TBSA ≥ 27% has the most impact on early intubation, and combined with BWT ≥ 3.5 mm, the intubation rate rose to 100%. Only one patient was intubated without TBSA ≥ 27%, neck burn, or COHb ≥ 4.0% (Fig. 1). The patient was hoarse. Combined together, if patients suffered ≥27% TBSA burned and showed BWT ≥ 3.5 mm, the positive predictive values for early intubation was 1.00. If patients suffered relatively small burns without neck burn and their COHb levels were less than 4.0%, the negative predictive value for early intubation was 0.97 (Table 5).

**Table 4:** Positive and negative predictive values for early intubation in patients with inhalation injury

	Positive predictive value	Negative predictive value
TBSA ≥27%	0.56	0.94
Facial burns	0.54	0.75
Neck burns	0.66	0.73
Singed nasal hair	0.47	0.66
Hoarseness	0.46	0.59
Use of accessory respiratory muscles	1.00	0.61
Noisy breath sounds	0.67	0.61
BWT ≥3.5 mm	0.73	0.72
COHb ≥4.0%	0.63	0.74
Ls	0.87	0.68
Bs	0.82	0.64

Cut‐off values for continuous variables were determined by receiver operating characteristic curve analysis.

Bs, severe edema, ulcer, necrosis of the bronchus without evidence of blood flow (pale mucosa without visible vessels), bronchorrhea, bronchial obstruction (any or combination); BWT, bronchial wall thickness; COHb, carboxyhemoglobin levels; Ls, (i) epiglottic blister or bullae; (ii) swelling of arytenoid region extends to the aryepiglottic fold; (iii) the length of the center of the glottic space when the vocal cords open maximally is shorter than the width of the center of the arytenoid region; (iv) the vocal cords are obscured because the vestibular folds are swollen; (v) the glottis does not open sufficiently due to marked vocal cord swelling (any or combination); TBSA, total body surface area burned.

**Table 5:** Positive and negative predictive values for early intubation of patients with inhalation injury using combined factors

	Positive predictive value	Negative predictive value
TBSA ≥27% and BWT ≥3.5 mm	1.00	0.68
TBSA <27% and COHb <4.0% and neck burn (−)	0.50	0.97
Ls and/or Bs	0.82	0.64

Combinations of factors were determined by classification and regression tree analysis.

Bs, severe edema, ulcer, necrosis of the bronchus without evidence of blood flow (pale mucosa without visible vessels), bronchorrhea, bronchial obstruction (any or combination); BWT, bronchial wall thickness; COHb, carboxyhemoglobin levels; Ls, (i) epiglottic blister or bullae; (ii) swelling of arytenoid region extends to the aryepiglottic fold; (iii) the length of the center of the glottic space when the vocal cords open maximally is shorter than the width of the center of the arytenoid region; (iv) the vocal cords are obscured because the vestibular folds are swollen; (v) the glottis does not open sufficiently due to marked vocal cord swelling (any or combination); TBSA, total body surface area burned.

Next, we determined the relationships between the FOB findings of the upper and lower airway and the other findings to confirm our findings anatomically (Tables 6 and 7, respectively). The FOB findings above the glottis were related with noisy breath sounds, facial and neck burns, hoarseness, and COHb level (Table 6), and those below the glottis were related with noisy breath sounds, BWT, and COHb level (*P* < 0.05) (Table 7).

**Table 6:** Relationships between fiberoptic bronchoscopy findings of the upper airway and other findings in patients with inhalation injury

	L−	Lm	Ls	*P*‐value
	*n* = 9	*n* = 56	*n* = 15	
Facial burns	6	28	14	0.004
Neck burns	3	17	12	0.002
Singed nasal hair	6	35	10	0.940
Hoarseness	0	17	7	0.016
Use of accessory respiratory muscles	0	4	1	0.540
Noisy breath sounds	0	4	5	0.015
BWT, mm	3.1 (2.2, 3.2)	3.0 (2.6, 3.8)	3.5 (2.8, 4.2)	0.290
COHb, %	1.4 (0.2, 2.8)	3.1 (1.2, 9.0)	4.4 (2.2, 21.7)	0.020

Data are expressed as number of cases or median (interquartile range).

Relationships between fiberoptic bronchoscopy severities and categorical data were tested using Fisher's exact test and continuous variables were analyzed by the Kruskal–Wallis test.

BWT, bronchial wall thickness; COHb, carboxyhemoglobin levels; L−, absence of carbonaceous deposits, erythema, edema, bronchorrhea, or obstruction; Lm, minor or patchy areas of erythema, carbonaceous deposits in laryngeal area; Ls, (i) epiglottic blister or bullae; (ii) swelling of arytenoid region extends to the aryepiglottic fold; (iii) the length of the center of the glottic space when the vocal cords open maximally is shorter than the width of the center of the arytenoid region; (iv) the vocal cords are obscured because the vestibular folds are swollen; (v) the glottis does not open sufficiently due to marked vocal cord swelling (any or combination).

**Table 7:** Relationships between fiberoptic bronchoscopy findings of the lower airway and other findings in patients with inhalation injury

	B−	Bm	Bs	*P*‐value
	*n* = 30	*n* = 39	*n* = 11	
Facial burns	20	22	6	0.3100
Neck burns	13	15	4	0.2800
Singed nasal hair	22	20	9	0.2100
Hoarseness	6	13	5	0.3000
Use of accessory respiratory muscles	1	2	2	0.1700
Noisy breath sounds	1	4	4	0.0070
BWT, mm	3.0 (2.6, 3.6)	3.0 (2.5, 3.5)	4.1 (3.5, 5.2)	0.0100
COHb, %	1.9 (0.8, 2.8)	3.9 (1.5, 10)	23.3 (9.1, 46.3)	<0.0001

Relationships between fiberoptic bronchoscopy severities and categorical data were tested using Fisher's exact test and continuous variables were analyzed by Kruskal–Wallis test.

Data are expressed as number of cases or median (interquartile range).

B−, absence of carbonaceous deposits, erythema, edema, bronchorrhea, or obstruction; Bm, hyperemia and/or edema of the bronchus with evidence of blood flow (presence of visible vessels through the mucosa); Bs, severe edema, ulcer, necrosis of the bronchus without evidence of blood flow (pale mucosa without visible vessels), bronchorrhea, bronchial obstruction (any or combination); BWT, bronchial wall thickness; COHb, carboxyhemoglobin levels.

## Discussion

In this study, we clearly showed that patients’ symptoms, COHb levels, and BWT were reliable parameters to predict early intubation, not singed nasal hair. We also showed the relationships between these findings and FOB findings. These findings may not be surprising for burn physicians. It is critical to predict those patients with inhalation injury who will require intubation. However, unnecessary intubation causes patients to suffer, and sedation and postural restriction cause expectoration of sputum that may lead to respiratory infection.^21^

Bronchoscopic diagnosis is generally accepted for inhalation injury.^17^ The Association for the Advancement of Automotive Medicine provided the AIS in which inhalation injuries of the lower glottis are graded according to their severity. However, there are no unified standards for grading inhalation injuries above the glottis, and this is why we developed our original FOB grading system for inhalation injury. The most frequent reasons for intubation were severe respiratory injuries, followed by extensive burn injury. Nine patients were intubated because of severe upper airway inhalation injury, and another three patients were diagnosed several days after the day of injury. As expected, all three patients showed facial and neck burns at presentation, which suggests that more attention must be paid to inhalation injury above the glottis when patients have suffered facial and neck burns, even if the patients were initially diagnosed as having mild injury. For those patients with inhalation injury above the glottis, BWT did not relate with severity, and the physical findings of the patients were the most reliable predictors. Moreover, we found that singed nasal hair did not relate with the severity of inhalation injury and could not predict the need for intubation. Most of the studies reporting on inhalation injuries have focused on the trachea, bronchus, and lung parenchyma, and few reports have addressed acute upper airway inhalation injuries. Our data shows that FOB findings are related with hoarseness and noisy breath sounds.

Inhalation injuries to the bronchus are difficult to evaluate because of the grime and soot coating the airway. We wash out the soot coating with a small amount of saline solution under the FOB, so our grading scale does not need the assessment of carbonaceous deposits to evaluate the severity of inhalation injury. Yamamura *et al*.^22^ reported that the AIS grade was not related with the duration of intubation and could not predict the development of pneumonia, and they concluded that BWT might be a more useful tool for predicting the development of pneumonia. We measured BWT as one of the risk factors of intubation and found that it was associated with intubation as expected. We suppose that, as a non‐invasive examination, BWT could be an important factor that predicts the need for intubation.

In 1971, Vincenti *et al*.^23^ reported that patients who suffered smoke inhalation did not show apparent symptoms on the day of injury and proposed that blood gas examination could be useful in the diagnosis of their inhalation injury. Researchers reported that fibrobronchoscopy was useful to diagnose lower glottis injuries. They also suggested that blood gas analysis and xenon scintiphotography was also useful tool to diagnose the severity and anatomical spread of inhalation injuries because it was difficult to diagnose these by bronchoscopy.^24^, ^25^ As shown in Table 7, physical findings were not reliable parameters with which to diagnose lower glottis inhalation injury, so we suppose that other medical examinations are needed in the diagnosis, such as blood gas analysis and/or diagnostic imaging. Xenon scintiphotography could be useful, although it is not commonly used in all burn centers. More commonly available tools are absolutely needed. Moreover, to our knowledge, although there are no reports that distinguish imaging as a tool for the prediction of intubation, the CT scan can be useful. We propose that, in patients with inhalation injury, intubation should be considered if they show a BWT ≥3.5 mm and COHb level ≥4.0%.

In summary, for inhalation injuries above the glottis, physical findings are the important factors predictive of intubation; for inhalation injuries below the glottis, BWT and COHb are the useful predictive factors.

We have some limitations in this study. First, this was a single‐center, retrospective study, and some emergency physicians might forget to record the physical findings in medical charts. Second, we still do not know the absolute necessity of early intubation without urgent situations including shock, impaired oxygen, or coma. We could not extubate any patients within 24 h. Some unnecessary early intubation might induce fluid creep, which produces more edema formation around the glottis. However, we believe that our daily practice for early intubation is still acceptable in that we could avoid unnecessary intubation in patients with mild inhalation injury. Finally, the number of transfusions before diagnosis could be an important factor for early intubation. We definitely need prospective studies to reveal the relationships between fluid amount and early intubation.

## Conclusions

Our findings show that singed nasal hair cannot predict the need for early intubation in patients with inhalation injury. Use of accessory respiratory muscles, noisy breath sounds, BWT on chest CT images, and the COHb level are reliable factors to determine the indication for early intubation. We believe that these findings are valuable for ER physicians, especially those who are not familiar with burn patient care.

## Conflict of Interest

None DECLARED.

## References

[1] Mlcak RP , Suman OE , Herndon DN . Respiratory management of inhalation injury. Burns 2007; 33(1): 2–13. 17223484 10.1016/j.burns.2006.07.007

[2] Suzuki M , Aikawa N , Kobayashi K , Higuchi R . Prognostic implications of inhalation injury in burn patients in Tokyo. Burns 2005; 31(3): 331–6. 15774289 10.1016/j.burns.2004.10.016

[3] Dai NT , Chen TM , Cheng TY *et al* The comparison of early fluid therapy in extensive flame burns between inhalation and noninhalation injuries. Burns 1998; 24(7): 671–5. 9882069 10.1016/s0305-4179(98)00092-8

[4] Shirani KZ , Pruitt BA Jr , Mason AD Jr . The influence of inhalation injury and pneumonia on burn mortality. Ann. Surg. 1987; 205(1): 82–7. 3800465 10.1097/00000658-198701000-00015 PMC1492872

[5] Heimbach DM , Waeckerle JF . Inhalation injuries. Ann. Emerg. Med. 1988; 17(12): 1316–20. 3057948 10.1016/s0196-0644(88)80357-3

[6] Pruitt BA Jr , Cioffi WG , Shimazu T , Ikeuchi H , Mason AD Jr . Evaluation and management of patients with inhalation injury. J. Trauma 1990; 30(12 Suppl): S63–8. 2254994 10.1097/00005373-199012001-00015

[7] Park MS , Cancio LC , Batchinsky AI *et al* Assessment of severity of ovine smoke inhalation injury by analysis of computed tomographic scans. J. Trauma 2003; 55(3): 417–27; discussion 27‐9. 14501881 10.1097/01.TA.0000083609.24440.7F

[8] Cancio LC , Batchinsky AI , Dubick MA *et al* Inhalation injury: pathophysiology and clinical care proceedings of a symposium conducted at the Trauma Institute of San Antonio, San Antonio, TX, USA on 28 March 2006. Burns 2007; 33(6): 681–92. 17532146 10.1016/j.burns.2006.11.009

[9] Shimazu T , Yukioka T , Ikeuchi H , Mason AD Jr , Wagner PD , Pruitt BA Jr . Ventilation‐perfusion alterations after smoke inhalation injury in an ovine model. J. Appl. Physiol. 1996; 81(5): 2250–9. 8941552 10.1152/jappl.1996.81.5.2250

[10] Albright JM , Romero J , Saini V *et al* Proteasomes in human bronchoalveolar lavage fluid after burn and inhalation injury. J. Burn Care Res. 2009; 30(6): 948–56. 19826256 10.1097/BCR.0b013e3181c07f37 PMC3904667

[11] Kurzius‐Spencer M , Foster K , Littau S *et al* Tracheobronchial protease inhibitors, body surface area burns, and mortality in smoke inhalation. J. Burn Care Res. 2009; 30(5): 824–31. 19692916 10.1097/BCR.0b013e3181b47ee8 PMC4418516

[12] Kurzius‐Spencer M , Foster K , Littau S *et al* Tracheobronchial markers of lung injury in smoke inhalation victims. J. Burn Care Res. 2008; 29(2): 311–8. 18354287 10.1097/BCR.0b013e3181667991

[13] Endorf FW , Gamelli RL . Inhalation injury, pulmonary perturbations, and fluid resuscitation. J. Burn Care Res. 2007; 28(1): 80–3. 17211205 10.1097/BCR.0B013E31802C889F

[14] Mosier MJ , Gamelli RL , Halerz MM , Silver G . Microbial contamination in burn patients undergoing urgent intubation as part of their early airway management. J. Burn Care Res. 2008; 29(2): 304–10. 18354286 10.1097/BCR.0b013e318166daa5

[15] Woodson LC . Diagnosis and grading of inhalation injury. J. Burn Care Res. 2009; 30(1): 143–5. 19060739 10.1097/BCR.0b013e3181923b71

[16] Marek K , Piotr W , Stanislaw S *et al* Fibreoptic bronchoscopy in routine clinical practice in confirming the diagnosis and treatment of inhalation burns. Burns 2007; 33(5): 554–60. 17376597 10.1016/j.burns.2006.08.030

[17] Masanes MJ , Legendre C , Lioret N , Maillard D , Saizy R , Lebeau B . Fiberoptic bronchoscopy for the early diagnosis of subglottal inhalation injury: comparative value in the assessment of prognosis. J. Trauma 1994; 36(1): 59–67. 8295250 10.1097/00005373-199401000-00009

[18] Liffner G , Bak Z , Reske A , Sjoberg F . Inhalation injury assessed by score does not contribute to the development of acute respiratory distress syndrome in burn victims. Burns 2005; 31(3): 263–8. 15774279 10.1016/j.burns.2004.11.003

[19] Sullivan SR , Friedrich JB , Engrav LH *et al* “Opioid creep” is real and may be the cause of “fluid creep”. Burns 2004; 30(6): 583–90. 15302427 10.1016/j.burns.2004.03.002

[20] Inoue T , Sugiki D . Indications for Endotracheal Intubation in Patients with Inhalation Injury of the Larynx. JJAAM 2008; 19: 262–71.

[21] Levine SA , Niederman MS . The impact of tracheal intubation on host defenses and risks for nosocomial pneumonia. Clin. Chest Med. 1991; 12(3): 523–43. 1934953

[22] Yamamura H , Kaga S , Kaneda K , Mizobata Y . Chest computed tomography performed on admission helps predict the severity of smoke‐inhalation injury. Crit. Care 2013; 17(3): R95. 23706091 10.1186/cc12740 PMC3707034

[23] DiVincenti FC , Pruitt BA Jr , Reckler JM . Inhalation injuries. J. Trauma 1971; 11(2): 109–17. 5099825 10.1097/00005373-197102000-00002

[24] Hunt JL , Agee RN , Pruitt BA Jr . Fiberoptic bronchoscopy in acute inhalation injury. J. Trauma 1975; 15(8): 641–9. 1152086 10.1097/00005373-197508000-00004

[25] Agee RN , Long JM III , Hunt JL *et al* Use of 133xenon in early diagnosis of inhalation injury. J. Trauma 1976; 16(3): 218–24. 1255837 10.1097/00005373-197603000-00007

